# The Changing Landscape of Pharmacotherapy for Diabetes Mellitus: A Review of Cardiovascular Outcomes

**DOI:** 10.3390/ijms20235853

**Published:** 2019-11-21

**Authors:** Linda Wu, Jenny E. Gunton

**Affiliations:** 1 Department of Endocrinology, Royal North Shore Hospital, St Leonards, NSW 2065, Australia; 2 Centre for Diabetes, Obesity and Endocrinology, The Westmead Institute for Medical Research, The University of Sydney, Westmead, NSW 2145, Australia; jenny.gunton@sydney.edu.au; 3 Sydney Medical School, Faculty of Medicine and Health, The University of Sydney, Westmead, NSW 2145, Australia; 4 Garvan Institute of Medical Research, Darlinghurst, NSW 2145, Australia

**Keywords:** diabetes mellitus, cardiovascular risk, cardiovascular outcomes, glycaemic control, glucose lowering therapy

## Abstract

The prevention of cardiovascular morbidity and mortality has always been a primary concern in patients with type 2 diabetes. Modern trials of glucose-lowering therapies now assess major adverse cardiac events as an endpoint in addition to the effects on glycaemic control. Whilst the data on the efficacy of intensive glucose lowering on reducing cardiovascular risk are limited, there are now increasing numbers of glucose-lowering therapies that have proven cardiovascular benefit independent of glucose lowering. This review will summarise the available literature on cardiovascular outcomes in relation to metformin, sulphonylureas, di-peptidyl peptidase-4 inhibitors, glucagon-like peptide receptor agonists, sodium-glucose co-transporter 2 inhibitors, thiazolidinediones, acarbose and insulin. In addition, new paradigms in diabetes management and the importance of treatment selection based on considerations including but not limited to glycaemic control will be discussed.

## 1. Introduction

Cardiovascular disease is the leading cause of morbidity and mortality in patients with diabetes mellitus [1].

Although improving glycaemic control has been a longstanding focus in diabetes management, intensive glycaemic control alone was not proven to lead to a reduction in cardiovascular risk. The controversy about rosiglitazone and cardiovascular events led to the RECORD study [2] and the Food and Drug Administration (FDA) mandating cardiovascular outcome trials in diabetes. There is now an expanding repertoire of pharmacological agents for the treatment of type 2 diabetes (T2D) that not only demonstrate cardiovascular safety but also may reduce cardiovascular events. This review will focus on the commercially available pharmacotherapies for T2D and the impact on cardiovascular outcomes.

## 2. Pathophysiology and Spectrum of Diabetic Heart Disease

Diabetic heart disease encompasses a spectrum of disorders ranging from myocardial infarction (MI) to diabetic cardiomyopathy, heart failure and increased risk of sudden death. Studies report that cardiac failure is 2–8 fold more common in patients with diabetes, with diastolic dysfunction being more common than systolic dysfunction [3,4]. Diabetic cardiomyopathy refers to a specific form of heart disease independent of coronary artery disease and hypertension that arises as a result of damage to the myocardium from long-term hyperglycaemia [5]. Diastolic dysfunction and eccentric hypertrophy are common signs of diabetic cardiomyopathy [6].

**Figure 1:**
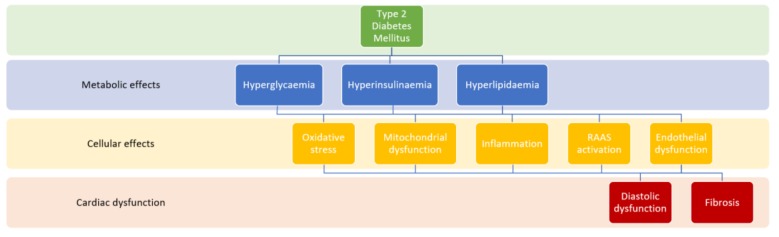
Pathophysiology of diabetic cardiomyopathy [5,6].

There are many mechanisms by which diabetes leads to diabetic heart disease (Figure 1). Diabetes leads to an increased risk of atherosclerosis of coronary vessels. Hyperglycaemia is thought to lead to increased inflammation via multiple mechanisms including increased advanced-glycation end products, cardiac lipotoxicity, activation of the renin–angiotensin–aldosterone system, abnormal coronary microcirculation, mitochondrial dysfunction and impaired calcium handling [5,6]. Prolonged inflammation is implicated in the development of myocardial hypertrophy, fibrosis and subsequent diastolic dysfunction [6]. Insulin resistance independently predisposes to the development of diabetic cardiomyopathy; however, its precise role in its pathogenesis is yet to be determined [7].

## 3. Pharmacotherapy for Diabetes

### 3.1. Metformin

Metformin is the most prescribed oral hypoglycaemic agent worldwide and is generally considered first-line therapy in T2D [8]. Metformin is a biguanide that decreases hepatic gluconeogenesis and increases peripheral glucose utilisation [9]. It also improves dyslipidaemia and has anti-inflammatory effects. It leads to an approximate reduction in glycosylated haemoglobin (HbA1c) by 1.1–1.5% [10]. It is generally well tolerated, and its side effects include gastrointestinal upset and, rarely, lactic acidosis [9].

As metformin has been on the market for more than 60 years, it predates the advent of cardiovascular outcome trials, and limited randomised controlled trial (RCT) data are available. A recent meta-analysis of 13 studies of metformin in T2D concluded that metformin appeared to be safe, but no significant reduction in cardiovascular risk could be concluded [11]. The largest study amongst those analysed was the UKPDS trial, which included a substudy of 753 obese patients who were randomised to receive metformin, sulphonylurea, insulin or conventional diet therapy [12]. Metformin was associated with a 32% risk reduction for any diabetes-related endpoint, including both macrovascular and microvascular diseases. Metformin also resulted in a significant reduction of myocardial infarction (39%), diabetes-related death (42%) and all-cause mortality (36%).

Of the other RCTs involving metformin, two analysed cardiovascular outcomes. Kooy et al. analysed 390 patients with T2D treated with either metformin or placebo and found no significant difference in the primary endpoint of combined macrovascular and microvascular morbidity and mortality [13]. There was a significant reduction in the macrovascular endpoint (*p* = 0.02), which was attributed in part to the difference in weight between groups. Hong et al. studied 304 patients with T2D randomised to receive either glipizide or metformin [14]. Similar to the UKPDS study, metformin had a lower risk of composite cardiovascular events compared to sulphonylureas (HR = 0.54, *p* = 0.02).

Overall, from the limited data available, it can be concluded that metformin does not lead to excess cardiovascular risk and is likely to reduce cardiovascular risk.

### 3.2. Sodium–Glucose Cotransporter 2 (SGLT2) Inhibitors

SGLT2 inhibitors were the first class of glucose-lowering agents demonstrated to have unequivocal cardiovascular benefit. The introduction of these new agents has drastically altered the clinical practice, with reduction in risk of heart failure exacerbation, cardiovascular death, and progression to renal failure observed with canagliflozin and empagliflozin administration [15,16]. Unexpectedly, dapagliflozin did not reduce the primary major adverse cardiovascular events (MACE) rate, although it was beneficial for both heart failure and renal disfunction progression [17]. Trials are currently running for ertugliflozin (completion 2020) and sotagliflozin (completion 2019).

SGLT2 inhibitors block the resorption of glucose in the proximal renal tubule via the SGLT2 transporter and thus promote glycosuria with consequent diuresis and natriuresis [18]. In addition, SGLT2 inhibitors are associated with approximately 2 kg weight loss, reduced systolic blood pressure (3 mmHg) and reduction in HbA1c by approximately 0.5–0.7% [19]. The main side effects include increased risk of dehydration and acute renal impairment, genital infections, urinary incontinence and euglycaemic ketoacidosis [19]. An association with increased risk of lower limb amputations was found in the canagliflozin trial [15]. This was not demonstrated in subsequent canagliflozin trials or other SGLT2 inhibitor trials

**Figure 2:**
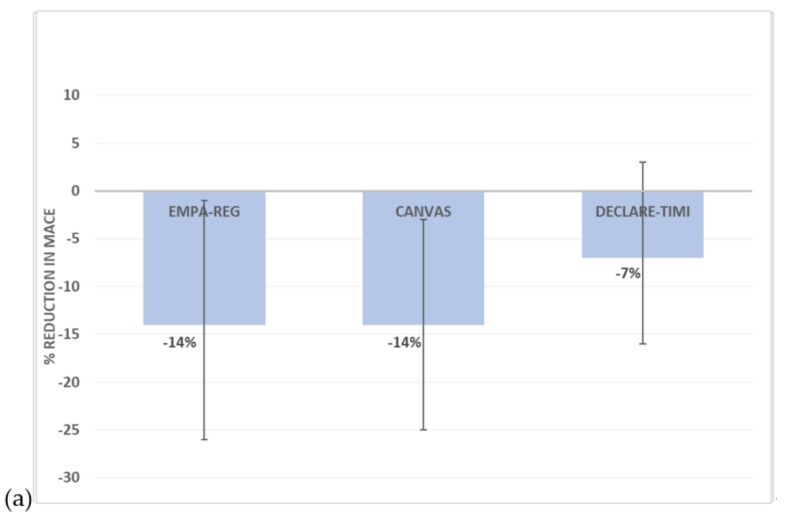

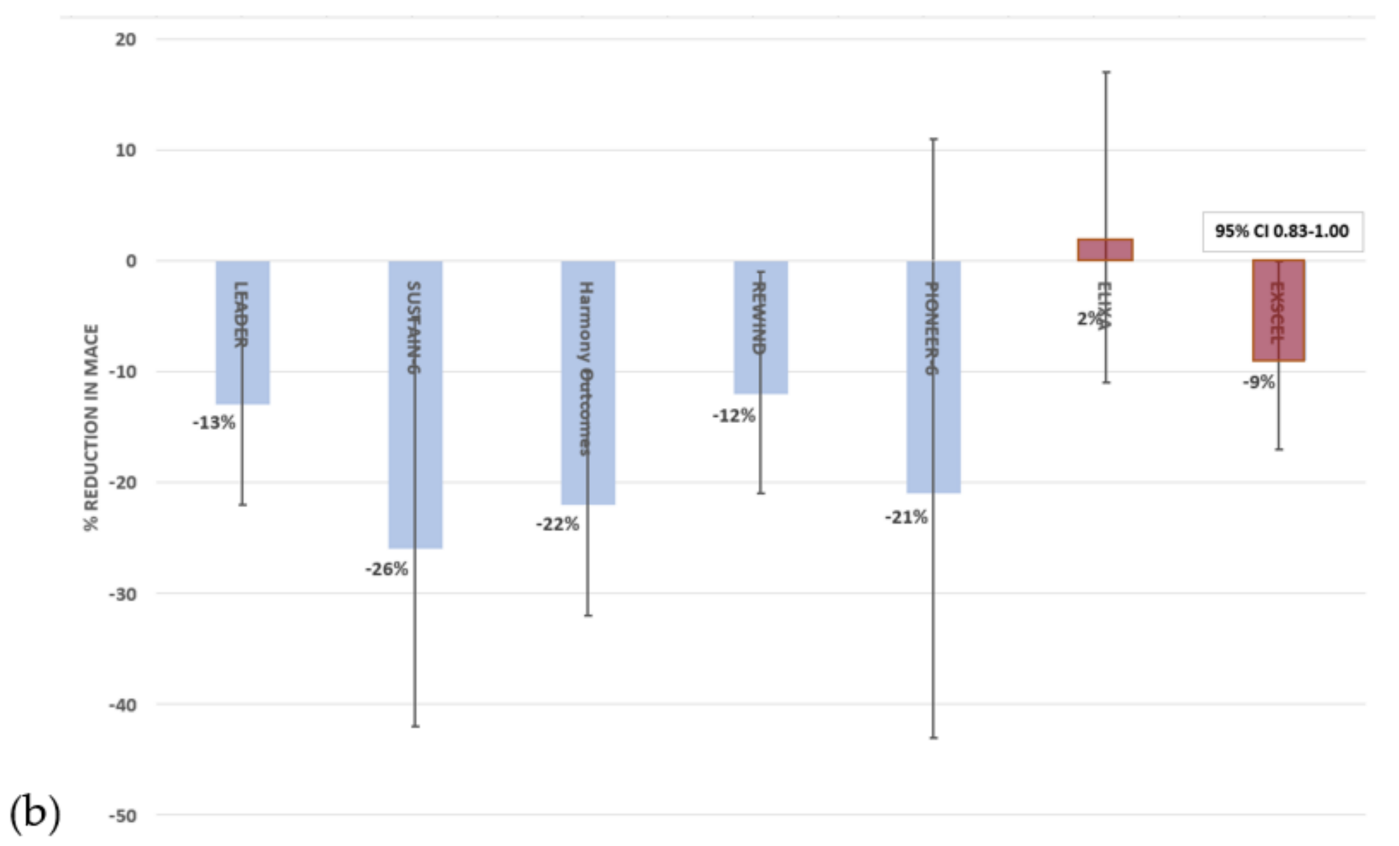
Summary of effects on major adverse cardiovascular outcomes including death from cardiovascular causes, non-fatal myocardial infarction and non-fatal stroke for (**a**) Sodium–glucose cotransporter 2 (SGLT2) inhibitors and (**b**) Glucagon-like peptide 1 receptor agonists (GLP1-RA) in patients with type 2 diabetes (blue = human GLP1-based, red = exendin-4 based). Error bars represent 95% confidence intervals.

Currently, there are four FDA-approved SGLT2 inhibitors, i.e., empagliflozin, canagliflozin, dapagliflozin and ertugliflozin. Figure 2a summarises the cardiovascular outcomes from the trials released thus far. In the landmark Empagliflozin Cardiovascular Outcome Event (EMPA-REG Outcome) trial of 7020 patients with high cardiovascular risk, empagliflozin was the first SGLT2 inhibitor demonstrating a significant reduction in the 3-point MACE (3P-MACE) of cardiovascular death, non-fatal myocardial infarction and non-fatal stroke (HR = 0.86, 95% CI 0.74–0.99) [16]. There was also a 35% reduction in hospitalisation for heart failure, a 38% reduction in cardiovascular mortality and a 32% reduction in all-cause mortality. These benefits were noted early with separation of curves within 1 year and were present with both a low dose (10 mg) and a standard dose (25 mg).

These benefits were similarly noted in the Canagliflozin Cardiovascular Assessment (CANVAS) trial where canagliflozin significantly reduced MACE compared to placebo (HR = 0.86, 95% CI 0.75–0.97) and reduced heart failure hospitalisation (HR = 0.67, 95% CI 0.52–0.87) [15]. In contrast to the EMPA-REG trial where >99% patients had established cardiovascular disease (CVD), the CANVAS trial included patients with and without CVD, and the beneficial effect of canagliflozin was found to be predominantly in secondary prevention, with a nonsignificant hazard ratio of 0.98 in the primary prevention group.

Interestingly, in the recently published DECLARE-TIMI 58 trial, dapagliflozin is the only SGLT2 inhibitor to date to not cause a significant reduction in 3-point MACE (HR = 0.93, 95% CI 0.84–1.03, *p* = 0.17). It did lead to a lower risk of hospitalisation for heart failure (HR = 0.73, 95% CI 0.61–0.88), with no difference in cardiovascular death (HR = 0.98, 95% CI 0.82–1.17) [17]. This was hypothesised to relate to the exclusion of patients with creatinine clearance <60 mL/min in comparison to the previous trials. This may exclude a population with chronic kidney disease that potentially stand to gain larger mortality benefit due to the additional renoprotective effects of SGLT2 inhibitors. However, DECLARE-TIMI randomized >17,000 people, and there was no trend for benefit in the secondary prevention group.

Perhaps the most practice-changing of trials in glucose-lowering medications will be the recently published DAPA-HF trial of dapagliflozin in 4744 patients with class II–IV New York Heart Association (NYHA) heart failure and ejection fraction ≤40% both with and without diabetes [20]. Over a median of 18.2 months, the primary outcome of worsening heart failure or cardiovascular death was reduced by 26% (HR = 0.74, 95% CI 0.65–0.85), with a significant reduction in worsening heart failure (HR = 0.7, 95% CI 0.59–0.83) and in cardiovascular death (HR = 0.82, 95% CI 0.71–0.97). These outcomes were not significantly different between patients with or without diabetes, and the incidence of side effects including hypoglycaemia were not significantly different between groups. These benefits were greater in the group with NYHA class II symptoms (HR = 0.63, 95% CI 0.52–0.75) compared to those with NYHA III or IV symptoms (HR = 0.9, 95% CI 0.74–1.09). The incidence of euglycaemic ketoacidosis was 0.1%, that of major hypoglycaemia was 0.5%, and both events only occurred in those with diabetes. This trial is the first to demonstrate reduced mortality in a group without diabetes with the use of glucose-lowering therapy and is likely to expand the use of SGLT2 inhibitors into the general management of heart failure with a reduced ejection fraction. The ongoing EMPEROR-Reduced and EMPEROR-Preserved trials will clarify if empagliflozin has similar efficacy in patients with and without diabetes, with heart failure with reduced and preserved ejection fraction, respectively.

Since the advent of these above-mentioned trials, the underlying mechanism of cardiovascular benefit has been extensively discussed but not yet definitively proven. There is evidence that SGLT2 inhibitors lead to improvement in ventricular loading, with preload reduced via diuresis and natriuresis and afterload reduced via blood pressure reduction and improved vascular function [21]. In the EMPA-REG OUTCOME trial, 50% of the cardiovascular benefit was attributed to haemoconcentration [22], and further trials have suggested that in comparison to standard diuretic therapy, SGLT2 inhibitors may deplete interstitial rather than intravascular fluid as a potential rationale for benefit [23,24]. Although there is yet to be any definitive evidence that SGLT2 inhibitors are beneficial for myocardial energetics, many have postulated the potential beneficial role of ketones such as beta-hydroxy-butyrate as an alternative myocardial fuel source [25]. There is also some evidence that SGLT2 inhibitors may promote branched-chain amino acid degradation, a process which is known to be impaired in heart failure [26]. Empagliflozin, canagliflozin and dapagliflozin have also been shown to inhibit Na^+^/H^+^ exchanger (NHE) 1 in the myocardium in addition to NHE 3 in the proximal tubule, suggesting a class effect [27]. NHEs have been shown to have increased expression in diabetes-associated heart failure, and inhibition of NHEs can reduce cytoplasmic sodium and calcium and may have a role in reducing heart failure risk via a common cardio–renal mechanism [28]. Additional studies have looked into the antifibrotic effects of SGLT2 inhibitors [29] and the effects on adipokine production [30]. The EMPA-HEART study that is currently underway will help to clarify the effects of empagliflozin on left ventricular mass and remodelling via cardiac MRI. Finally, there is emerging evidence that SGLT2 inhibitors can restore the disrupted circadian rhythm of blood pressure to produce a more pronounced nocturnal ‘dip’ in blood pressure [31]. The timing of antihypertensives to fit with the circadian rhythm has been demonstrated to lead to cardiovascular benefits, and this may explain part of the beneficial effects of SGLT2 inhibitors [32].

The renoprotective effects of SGLT2 inhibitors are also key in the management of diabetic nephropathy and chronic kidney disease and likely also contribute to cardiovascular benefit. Natriuresis leads to enhanced tubuloglomerular feedback and afferent arteriolar vasoconstriction which is thought to be key in long-term renal protection [33]. The EMPA-REG outcome trial demonstrated a significantly lowered risk of renal replacement therapy (RRT) but no difference in microalbuminuria, whilst the CANVAS trial found a significantly reduced incidence of microalbuminuria and no difference in the need for RRT [15,16]. The DECLARE-TIMI 58 trial also found a significant reduction in a composite endpoint of 40% decrease in estimated glomerular filtration rate (eGFR), end stage renal disease and renal death in comparison to placebo [17]. It is worth noting that in these trials, renal outcomes were secondary endpoints. The Canagliflozin and Renal Outcomes in Type 2 Diabetes and Nephropathy (CREDENCE) trial was the first to demonstrate a 34% reduction in the renal specific outcome of end-stage kidney disease, doubling of serum creatinine or death from renal causes in comparison to placebo (HR =0.70, 95% CI 0.59–0.82) [34]. Similar trials are underway to investigate the renoprotective effects of empagliflozin (EMPA-KIDNEY) and dapagliflozin (Dapa-CKD).

### 3.3. Glucagon-Like Peptide 1 Receptor Agonists

Glucagon-like peptide 1 (GLP1) receptor agonists (GLP1-RA) increase the effect of GLP-1, which is an incretin secreted in response to eating. This has the effect of increasing glucose-dependent insulin secretion, reducing glucagon secretion, slowing gastric emptying and suppressing appetite [35]. This class of medication is not only appealing for its glucose-lowering effects (HbA1c reduction 0.8–1.6%) but also effective for weight loss, lowering the risk of hypoglycaemia and inducing small improvements in blood pressure and lipid profiles [35].

GLP1-RA are heterogenous in structure, and dosing interval and efficacy and cardiovascular effects also vary (Figure 2b). Of the seven GLP1-RA studied, lixisenatide and exenatide are structurally based on exendin-4, whereas dulaglutide, albiglutide, liraglutide and semaglutide are based on human GLP-1. The overall efficacy of GLP1-RA in the reduction of 3P-MACE was 12% in a recent meta-analysis of the seven RCTs published to date [36].

Several human GLP1-based medications have demonstrated superiority over placebo for cardiovascular risk. As shown in Figure 2, reduction in 3P-MACE was demonstrated in the LEADER trial of liraglutide (hazard ratio (HR) = 0.87, 95% CI 0.78–0.97) [37], SUSTAIN-6 trial of subcutaneous semaglutide (HR = 0.74, 95% CI 0.58–0.95) [38], Harmony Outcomes Trial of albiglutide (HR = 0.78, 95% CI 0.68–0.90) [39] and REWIND trial of dulaglutide (HR = 0.88, 95% CI 0.79–0.99) [40]. The PIONEER-6 Trial of oral semaglutide showed a nonsignificant reduction in 3P-MACE (HR = 0.79, 95% CI 0.57–1.11) but a significant reduction in cardiovascular death (HR = 0.49, 95% CI 0.27–0.92) [41].

Contrastingly, exendin-4-based agents have not demonstrated any cardiovascular benefits in trials to date. Whilst most GLP1RA are considered long-acting, lixisenatide was the only short-acting GLP1-RA and was assessed in the Evaluation of Lixisenatide in Acute Coronary Syndrome (ELIXA) trial [42]. This involved patients with T2D and recent (MI) and demonstrated neutral cardiovascular outcomes (HR = 1.02, 95% CI 0.89–1.17). The EXSCEL study of weekly exenatide (Bydureon^®^)—another exendin-4-based medication—also found no significant effect on 3P-MACE (HR = 0.91, 95 CI% 0.83–1.00) [43]. However, this trial had a discontinuation rate of 40% and a higher proportion of patients without previous cardiovascular disease. A recent meta-analysis failed to find a significant difference between exendin-4- and native GLP1-based agents [36]. The upcoming trial of efpeglenatide (AMPLITUDE-O trial), a long-acting exendin-4-based GLP1-RA will help clarify if cardiovascular benefit with GLP1-RA is associated with native GLP1- over exendin-4-based treatments.

A composite renal outcome of new-onset or persistent macroalbuminuria, persistent doubling of serum creatinine and eGFR ≤ 45 mL/min/1.73m^2^, need for RRT, and renal death was reduced with liraglutide (HR = 0.78, 95% CI 0.67–0.92), semaglutide (HR = 0.64, 95% CI 0.46–0.88) and dulaglutide (HR = 0.85, 95% CI 0.77–0.93) [37,38,40]. The AWARD-7 Study compared insulin glargine to dulaglutide in patients with T2D and stage 3–4 chronic kidney disease (CKD) and found similar effects on glycaemic control and a significantly smaller decline in eGFR with dulaglutide use [44].

Common side effects of GLP1-RA include gastrointestinal side effects, in particular nausea, vomiting and injection site reactions. Pancreatitis may be increased with GLP1-RA, although this is controversial, perhaps due to the increase in the background rate of pancreatitis in people with T2D. The cardiovascular RCTs have not shown a significant increase of pancreatitis, although those patients are not at marked risk of pancreatitis. Some trials found an increase in the incidence of medullary thyroid cancer. For those reasons, we would usually avoid GLP1-RA in people with medullary thyroid cancer and with a personal history of pancreatitis. Some studies have shown significant worsening of retinopathy, but this effect has not been consistently evaluated in trials and demonstrated as a class effect in meta-analysis [36]. The increased rate of retinopathy observed in some studies may related to the significant glucose-lowering effects of these medications [38]. The upcoming FOCUS trial of semaglutide will help clarify the risk of worsening retinopathy with GLP1-RA therapy.

### 3.4. Sulphonylureas

The role of sulphonylurea therapy in the treatment of T2D is controversial. Due to efficacy and cost, sulphonylureas such as gliclazide and glimepiride remain amongst the most commonly prescribed oral hypoglycaemic agents. Sulphonylureas bind to the sulphonylurea receptor on pancreatic beta cells and inhibit the potassium flux via ATP-dependent potassium channels, leading ultimately to cell depolarisation and insulin secretion. A recent systematic review of 31 RCTs found that over a median of 16 weeks, sulphonylurea therapy lowered HbA1c by 1.51% compared to placebo [45].

Major side-effects associated with sulphonylurea therapy include hypoglycaemia and weight gain, both having implications for cardiovascular health. Multiple systematic reviews have concurred that hypoglycaemia risk is increased with sulphonylurea therapy, with gliclazide associated with the lowest hypoglycaemia risk in one review [46], and glibenclamide with the highest hypoglycaemia risk in another [47]. Recent studies have suggested that genetic polymorphisms in the enzyme CYP2C9 is associated with an increased risk of hypoglycaemia with sulphonylurea treatment [48]. The UKPDS study found a median 4 kg weight gain in the three years after commencement of glibenclamide [12].

The cardiovascular safety of sulphonylurea therapy has been questioned, not only in light of the risk of hypoglycaemia and weight gain, but also due to early trial data. The University Group Diabetes Program study in 1970 was terminated early due to excessive mortality that did not reach statistical significance in the sulphonylurea-treated group and led to tolbutamide being withdrawn from the market [49]. This study has subsequently been criticised for flaws in design and statistical analysis [50]. A recent meta-analysis has concluded that the cardiovascular risk was not significantly different upon treatment with sulphonylureas or any of the other eight available glucose-lowering drugs [51]. A subsequent meta-analysis of 82 RCTs and 26 observational studies found a significantly increased risk of all-cause mortality (HR = 1.26, 95% CI 1.1–1.44) and cardiovascular-related mortality (HR = 1.46, 95% CI 1.21–1.77) compared to other glucose-lowering treatments [52].

Given the mixed data on cardiovascular harm with sulphonylureas with no evidence of benefit, for people with pre-existing renal or cardiovascular disease, an SGLT2 inhibitor or GLP1-RA should be considered. This short-term acceleration followed by long-term improved risk is a well-recognised complication of tightening glucose control, first demonstrated in type 1 diabetes with the Diabetes Control and Complications Trial [53].

### 3.5. Dipeptidyl Peptidase 4 Inhibitors

Dipeptidyl peptidase 4 (DPP4) inhibitors enhance insulin secretion and suppress glucagon secretion by preventing the degradation of incretins including GLP1 and GIP. Although having a modest glucose-lowering effect of reducing HbA1c by 0.7%, these agents are widely used as add-on therapy due to good tolerability, minimal effect on weight and low risk of hypoglycaemia [54]. Side effects associated with DPP4 inhibitors include nausea and vomiting and potentially a small increase in the risk of pancreatitis [54].

Trials involving DPP4 inhibitors have shown a neutral effect on cardiovascular risk, with one trial finding an increased risk of heart failure. The Saxagliptin Assessment of Vascular Outcomes Recorded in Patients with Diabetes Mellitus-Thrombolysis in MI trial (SAVOR-TIMI 53) showed no significant difference in primary MACE in a population with T2D and a high risk of cardiovascular events (HR = 1.00, 95% CI 0.89–1.12) [55]. There was, however, a significant increase in the risk of heart failure (HR = 1.27, 95% CI 1.07–1.51). The Examination of Cardiovascular Outcomes with Alogliptin versus Standard of Care Trial (EXAMINE) also found no significant difference in MACE (HR = 0.96), and a non-significant difference in heart failure hospitalisation (HR = 1.07; 95% CI 0.79–1.46) [56]. The Trial Evaluating Cardiovascular Outcomes in Sitagliptin (TECOS) found no difference in a composite primary outcome of MACE and unstable angina (HR = 0.98, 95% CI 0.88–1.09) and no difference in hospitalisation for heart failure (HR = 1.00, 95% CI 0.83–1.20) [57].

The Cardiovascular and Renal Microvascular Outcome Study with Linagliptin in Patients with Type 2 Diabetes Mellitus (CARMELINA) Trial compared linagliptin to placebo in those with high cardiovascular risk and chronic kidney disease. It found no significant difference in the primary MACE outcome (HR 1.02; 95% CI 0.89–1.17) or the secondary outcomes of death due to renal failure, end-stage renal disease or sustained ≥40% decrease in eGFR (HR 1.04, 95% CI 0.89–1.22) [58]. The yet-to-be-published CAROLINA trial comparing linagliptin to glimepiride use has also demonstrated non-inferiority, with no significant difference in 3P-MACE and a 77% reduction in hypoglycaemia [59].

In summary, DPP4 inhibitors cause 0.5–0.7% decrease in HbA1c and are a well-tolerated option with a neutral cardiovascular profile, except for increased heart failure incidence with saxagliptin.

### 3.6. Thiazolidinediones

Thiazolidinediones (TZDs) are synthetic ligands developed as activators of peroxisome proliferator-activated receptors-gamma (PPAR-gamma) which causes increased peripheral insulin sensitivity, increased adipogenesis and reduced endogenous glucose production [60]. Pioglitazone has strong PPAR-gamma and weaker PPAR-alpha activity, whereas rosiglitazone is a pure PPAR-gamma agonist. Both are associated with 1% reduction in HbA1c compared to placebo [61]. However, due to their differing PPAR activity, their effects on lipids are also variable, with pioglitazone being associated with reduced triglycerides, higher high density lipoprotein (HDL) levels and neutral effect on low density lipoprotein (LDL) [62]. Rosiglitazone is associated with increased levels of LDL and HDL [62].

The most common side effects of TZDs include weight gain (2–6 kg) and fluid retention [61]. Weight gain with TZD use is likely to related directly to agonism of PPAR-gamma receptors in the adipose tissue, and fluid retention may account for up to 75% of weight gain [63,64]. Fluid retention may relate to increased vascular permeability and increased expression of *ENaC* in the renal collecting tubules due to PPAR-gamma agonism [65]. Although early TZDs such as troglitazone were associated with hepatotoxicity, evidence has since shown safety and potential beneficial effects on liver function with both pioglitazone and rosiglitazone [66,67]. TZDs have been associated with increased fracture risk in women but not men [68,69]. Several studies have reported an increased incidence of bladder cancer with pioglitazone use [70,71]; however, more prospective data are needed to further define this risk.

Thiazolidinediones are amongst the most controversial of glucose-lowering agents in terms of cardiovascular risk. The initial meta-analysis of rosiglitazone involved 42 trials and found a significant OR of 1.43 for MI (95% CI 1.03–1.98) and a nonsignificant OR of 1.64 for cardiovascular (CV)-related death [72]. This subsequently triggered the FDA to require investigators to demonstrate cardiovascular safety for all subsequent new glucose-lowering agents. This meta-analysis has been criticised for its methodology, including lack of statistical homogeneity and inclusion of non-diabetic subjects [73]. In response to this controversy, the Rosiglitazone Evaluated for Cardiac Outcomes and Regulation of Glycaemia in Diabetes (RECORD) study was designed [2]. This was an RCT with 4447 patients and found no increase in overall cardiovascular risk, but an increased risk of fatal and non-fatal heart failure in those treated with rosiglitazone (HR = 2.10, 95% CI 1.35–3.27). The mechanism is likely to relate to fluid retention as discussed above, and TZDs are contraindicated in patients with heart failure (NYHA class III or IV). Some critics feel that an effect on cardiovascular outcomes may have been masked in the RECORD trial due to lower-than-anticipated event rates, poor adherence to medication and imbalances in the use of other medications with cardiovascular benefit such as statin therapy. Contrastingly, pioglitazone was found in a meta-analysis of 19 studies to have a non-significant association with reduced MI (HR = 0.81, 95% CI 0.64–1.02) and a significant reduction in the primary composite outcome of death, non-fatal MI and non-fatal stroke (HR = 0.82, 95% CI 0.72–0.94) [74]. Increased incidence of heart failure with the use of pioglitazone was still noted.

In subsequent publications, there remain conflicting data as to the cardiovascular safety of both pioglitazone and rosiglitazone, with multiple trials reporting increased risk of MI, and several reporting no significant effect [75,76,77]. In light of this, the rate of use of TZDs has significantly declined. The recent Insulin Resistance Intervention after Stroke trial showed a 24% reduction in the rate of the primary composite outcome of fatal/nonfatal MI or stroke in those treated with pioglitazone versus placebo in insulin-resistant but non-diabetic patients with recent stroke or transient ischaemic attack (HR = 0.76, 95% CI 0.62–0.93) [78]. A 25% reduction in acute coronary syndromes, a 50% reduction in MI and a 25% reduction in stroke was also noted. No effect on heart failure was noted, although patients with heart failure were excluded from this trial.

### 3.7. Acarbose

Acarbose is an alpha-glucosidase inhibitor that acts by delaying carbohydrate absorption at the level of the gut and reduces postprandial hyperglycaemia.

A meta-analysis of seven RCTs found a reduced risk of composite CV events (including CV death, stroke, peripheral arterial disease, angina, MI, heart failure and revascularisation) (HR = 0.65, 95% CI 0.48–0.88), as well as a reduction in MI (HR = 0.36, 95% CI 0.16–0.80) [79]. A large cohort study involving 17,366 patients on acarbose and 230,023 patients on metformin found acarbose use was associated with a higher risk of CV events, heart failure and ischaemic stroke compared to metformin [80]. Given that metformin, as discussed earlier, is likely to have cardiovascular benefit, this result does not necessarily indicate that acarbose is associated with cardiovascular harm, although it suggests that metformin is superior.

The Acarbose Cardiovascular Evaluation (ACE trial) was a large RCT published recently with 6522 patients with cardiovascular disease and impaired glucose tolerance randomised to either acarbose or placebo [81]. Progression to diabetes was significantly reduced with the use of acarbose (HR = 0.82, 95% CI 0.71–0.94); however, no significant difference was seen in a composite primary outcome of cardiovascular death, fatal and non-fatal MI and fatal and non-fatal stroke (HR = 0.98, 95% CI 0.86–1.11).

The above data suggest that acarbose may be beneficial for CV risk.

### 3.8. Insulin

The effect of insulin on cardiovascular risk is difficult to analyse, as insulin use is confounded by poor glycaemic control, more advanced diabetes and subsequent increased risk of cardiovascular events. In animal models, insulin use is associated with atherosclerosis, with increased lipid synthesis and plaque formation [82,83]. In humans, this has not been definitively demonstrated. Several observational studies report that insulin use is associated with increased cardiovascular events and mortality [84]; however, this is confounded by the aforementioned issues of poorer glycaemic control and advanced diabetes.

The UKPDS trial showed no difference in CV events between groups treated with insulin versus sulphonylureas [12]. The Outcome Reduction with Initial Glargine Intervention (ORIGIN trial) studied 12,537 patients with CV risk factors and T2D, impaired fasting glucose or impaired glucose tolerance and randomised these patients to receive insulin glargine titrated to achieve a target fasting glucose <5.3 mmol/L or standard care over six years and found no difference in CV events in either group (HR = 1.02, 95% CI 0.94–1.11) [85].

## 4. Treatment Considerations

Achieving glycaemic targets for patients with diabetes should not be the sole consideration in management and needs to be weighed up with patient goals, risk of hypoglycaemia, age and comorbidities such as chronic kidney disease, obesity and heart failure

The Action to Control Cardiovascular risk in Diabetes (ACCORD) study was terminated early due to a higher rate of total and cardiovascular deaths in the group assigned to intensive therapy [86], so an overly tight control is not without risk.

The United Kingdom Prospective Diabetes Study (UKPDS) first found a significant reduction in MI rates in participants assigned to intensive therapy at 17 years follow-up, implying a legacy effect [12]. Contrastingly, the Action in Diabetes and Vascular Disease: Preterax and Diamicron MR Controlled Evaluation (ADVANCE) trial found no significant difference in a primary composite outcome of cardiovascular death, non-fatal MI or non-fatal stroke [87]. Similarly, the Veterans Affairs Diabetes Trial (VADT) did not show any difference in time to first occurrence of cardiovascular event or death between intensive- and standard-therapy groups [88]. Although the risk of major cardiovascular outcomes was reduced during the time of separation of glycated haemoglobin curves between the intensive-therapy and the standard-therapy group, this benefit was no longer present after equalisation of the glycated haemoglobin levels. Some factors contributing to these differing results include variations in glycaemic therapy between these trials, baseline glycaemic control, proportion achieving glycaemic targets with intensive control as well as frequency of hypoglycaemia.

With mixed evidence for intensive glycaemic control on cardiovascular disease, clinicians should not only target a HbA1c appropriate for a patient’s age, comorbidities and hypoglycaemia risk, but also address other considerations important for cardiovascular health, such as blood pressure, lipid profile and use of oral hypoglycaemics with the most optimal cardiovascular benefit.

**Figure 3:**
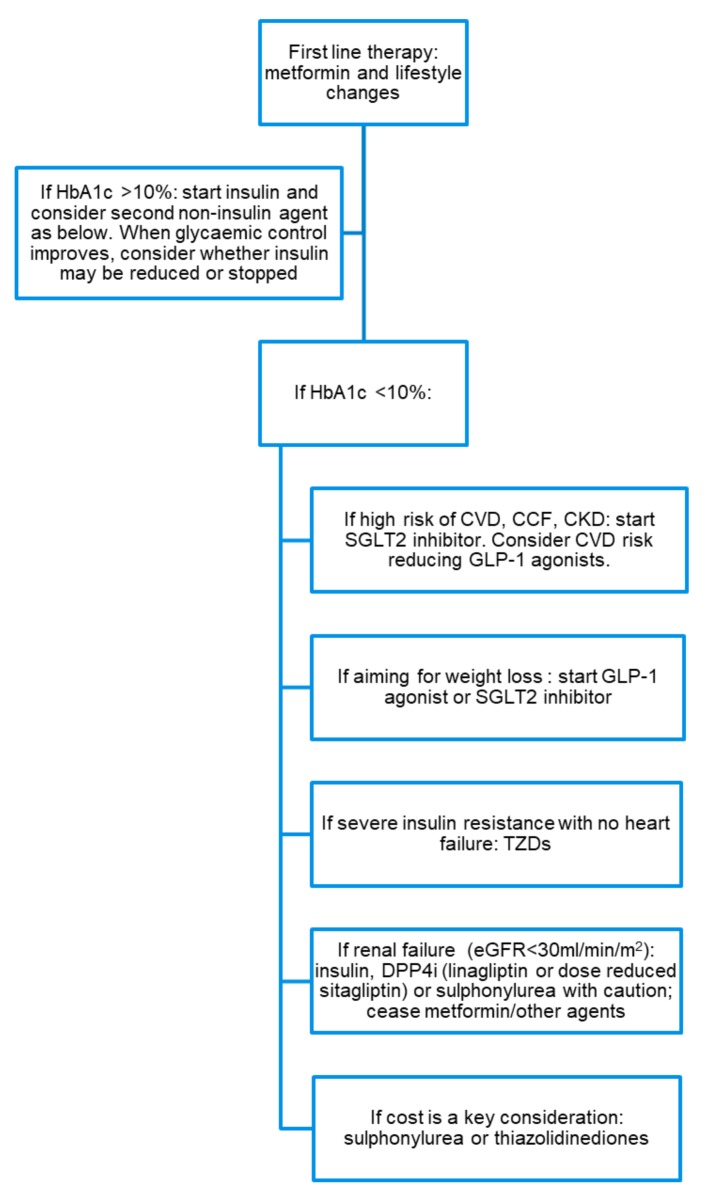
A flow diagram demonstrating a suggested approach to glucose-lowering therapies for type 2 diabetes (T2D). CVD: cardiovascular disease, CCF: congestive cardiac failure, CKD: chronic kidney disease, TZDs: thiazolidinediones, eGFR: estimated glomerular filtration rate.

Figure 3 depicts our suggested approach to the commencement of glucose-lowering therapies for patients with T2D. Metformin and lifestyle changes of weight control and dietary optimization remain key for most patients with T2D. The exceptions to this would be patients who are intolerant to metformin or have impaired renal function (eGFR < 30 mL/min/m^2^), for whom metformin use is contraindicated. Thereafter, there should be consideration as to whether insulin should be commenced upfront to ensure adequate glycaemic control, and our suggested threshold for consideration of immediate insulin would be HbA1c > 10%. We divide patients that require a second oral agent in addition to metformin to maintain glycaemic control on the basis of their key clinical characteristics. Patients who have a high cardiovascular risk, established CVD, heart failure with reduced ejection fraction, or diabetic nephropathy should be considered for SGLT2 inhibitor therapy unless contraindications exist. Obese individuals should be considered for GLP-1 agonist or SGLT2 inhibitor therapy. Patients who are severely insulin-resistant may be considered for thiazolidinedione therapy provided there is no history of heart failure. People with acanthosis nigricans would be especially suitable for TZDs in the absence of contraindications.

Finally, there are a select number of agents that are approved in stage IV–V CKD, namely, insulin, linagliptin, sitagliptin (at reduced dose) and some sulphonylureas.

Where cost is a major issue for patients, metformin and sulfonylureas are the cheapest agents, and acarbose is also a cost-effective option.

## 5. Future Directions

Gut microbiome dysbiosis has been found in several studies to be relate to progression of insulin resistance in diabetes and thus is a potential therapeutic target for the treatment of diabetes [89]. In mouse models, certain bacterial species significantly improve the symptom scores and reduce fat mass, endotoxaemia, tissue inflammation, as well as improve insulin secretion and sensitivity [90]. Similarly, prebiotics have also been found to enrich the gut microbiota, with associated improvements in intestinal permeability, reduction in inflammation and endotoxaemia and improvement in glucose tolerance [91]. Metformin, amongst the glucose-lowering therapies in particular, is associated with gut microbiome alterations, with increase in *Akkermansia muciniphilia* and increased species that produce butyrate and propionate, which are involved in glucose homeostasis [92].

With increasing evidence that chronotherapy, in the case of antihypertensive patients, has cardioprotective effects [32], similar circadian therapies may have promise for type 2 diabetes. Thus far, no trials have assessed the chronotherapeutic effects of glucose-lowering medications on cardiovascular or glycaemic outcomes in humans. Data are limited to mouse models, which have demonstrated certain novel clock-improving molecules to have benefits, with decrease in obesity and hyperglycaemia and improvement in insulin sensitivity [93].

As genetic testing becomes more readily available, more is now known regarding the effects of certain gene polymorphisms on pharmacokinetics and pharmacodynamics. For example, metformin is dependent on active transportation via the transporter OCT1 in order to enter the cell. Polymorphisms in the *SLC22A1* gene that codes for OCT1 have been shown to lead to variability in metformin efficacy and risk of intolerance [94]. Sulphonylureas rely on CYP2C9 for biotransformation in the liver, and variants in this enzyme have been reported to lead to reduced clearance of the drug [95]. The effects of polymorphisms on the cardiovascular benefits of diabetes medications remain to be elucidated.

Finally, there are numerous novel agents being investigated for the treatment of type 2 diabetes. Those that inhibit fatty acid oxidation carry theoretical potential for cardiovascular benefit. Fatty acid oxidation rates are increased in heart disease including diabetic cardiomyopathy and contribute to impaired cardiac function, as the use of fatty acids for ATP production reduces cardiac efficiency. Potential agents that inhibit fatty acid oxidation include CPT-1 inhibitors (e.g., perhexiline, etomoxir), mitochondrial fatty acid oxidation inhibitors (trimetazidine), malonyl CoA decarboxylase (MCD) inhibitors (CBM-301106), and pyruvate dehydrogenase kinase inhibitors (PDHK, e.g., dichloroacetate) [96]. Several PDHK inhibitors have demonstrated efficacy in lowering blood glucose levels in diabetic rodent models [97]. Trimetazidine has also been shown to improve cardiac function, reduce glycolysis and increase glucose oxidation, thereby reducing fatty acid oxidation [98].

## 6. Conclusions

The pharmacotherapeutic landscape of T2D has transformed from a dogma of optimal glucose lowering to one that recognizes that independent of glucose lowering, significant reduction in the risk of macrovascular complications of diabetes can be achieved. Surprising to all, agents such as SGLT2 inhibitors not only lower glucose but also have enhanced our understanding of the pathophysiology of heart failure. There is yet no randomised control trial data on the safety of these glucose-lowering agents in type 1 diabetes or cardiovascular outcomes, and this will need to be addressed in trials to come.
